# PRaG5.0 combined with chemotherapy and sequential chemoimmunotherapy for massive SMARCA4-deficient undifferentiated tumor of the cervix: case report

**DOI:** 10.3389/fimmu.2026.1826847

**Published:** 2026-05-08

**Authors:** Zixin Li, Xuezhou Pang, Zhongyao Wang, Jing Wen, Yanli Zhao, Daiyuan Ma, Jianquan Yang

**Affiliations:** 1 Department of Oncology, Affiliated Hospital of North Sichuan Medical College, Nanchong, Sichuan, China; 2 Department of Hepatobiliary Surgery, Affiliated Hospital of North Sichuan Medical College, Nanchong, Sichuan, China

**Keywords:** cadonilimab, cervical tumor, PRaG5.0, radiotherapy, SMARCA4

## Abstract

SMARCA4-deficient undifferentiated cervical tumor is highly malignant and responds poorly to conventional treatments, leading to a very poor prognosis. We report a case involving a primary cervical lesion measuring 9.8 cm, managed using a strategy of “PRaG5.0” combined with chemotherapy followed by sequential chemoimmunotherapy. This regimen employs short-course stereotactic body radiotherapy (SBRT, 24Gy/3Fx) as the radiotherapy component, combined with cadonilimab (a PD-1/CTLA-4 bispecific antibody), GM-CSF and thymosin α1 to form the PRaG5.0, alongside chemotherapy with nab-paclitaxel. Following the initial PRaG5.0 with chemotherapy, the patient’s symptoms were markedly relieved, and CT images showed the primary lesion had shrunk to 7.2×6.0 cm. Subsequent consolidation with chemoimmunotherapy further reduced the tumor to 3.7×2.9 cm, and the treatment was well-tolerated. The patient’s progression-free survival (PFS) now exceeds 12 months. This case indicates that for massive, refractory cervical tumors, PRaG5.0 combined with chemotherapy followed by sequential chemoimmunotherapy may offer an effective approach for controlling disease and extending survival.

## Introduction

Cervical cancer is one of the most common malignancies in women worldwide (1). SMARCA4-deficient undifferentiated cervical carcinoma is a rare subtype characterized by high aggressiveness, a propensity for recurrence and metastasis, poor response to conventional chemoradiotherapy, and the median survival time less than 6 months (2, 3). The management of massive cervical tumors (diameter ≥ 4 cm) presents a particular therapeutic challenge (4). This article reports a case of a patient presenting a massive SMARCA4-deficient undifferentiated cervical tumor, whose primary cervical lesion measured 9.8 cm in diameter, accompanied by vaginal bleeding and renal insufficiency. We adopted the “PRaG5.0 combined with chemotherapy and sequential chemoimmunotherapy regimen”. In this regimen, PRaG5.0 consists of short-course SBRT as the core of radiotherapy, combined with cadonilimab, GM-CSF, and thymosin α1 (5). Nab-paclitaxel chemotherapy is administered subsequently, followed by sequential chemoimmunotherapy. After this systemic treatment, the patient’s cervical lesions continued to shrink, and the efficacy evaluation was partial response (PR). At the last follow-up, the patient had achieved more than 12 months of PFS.

## Case presentation

**Figure 1:**
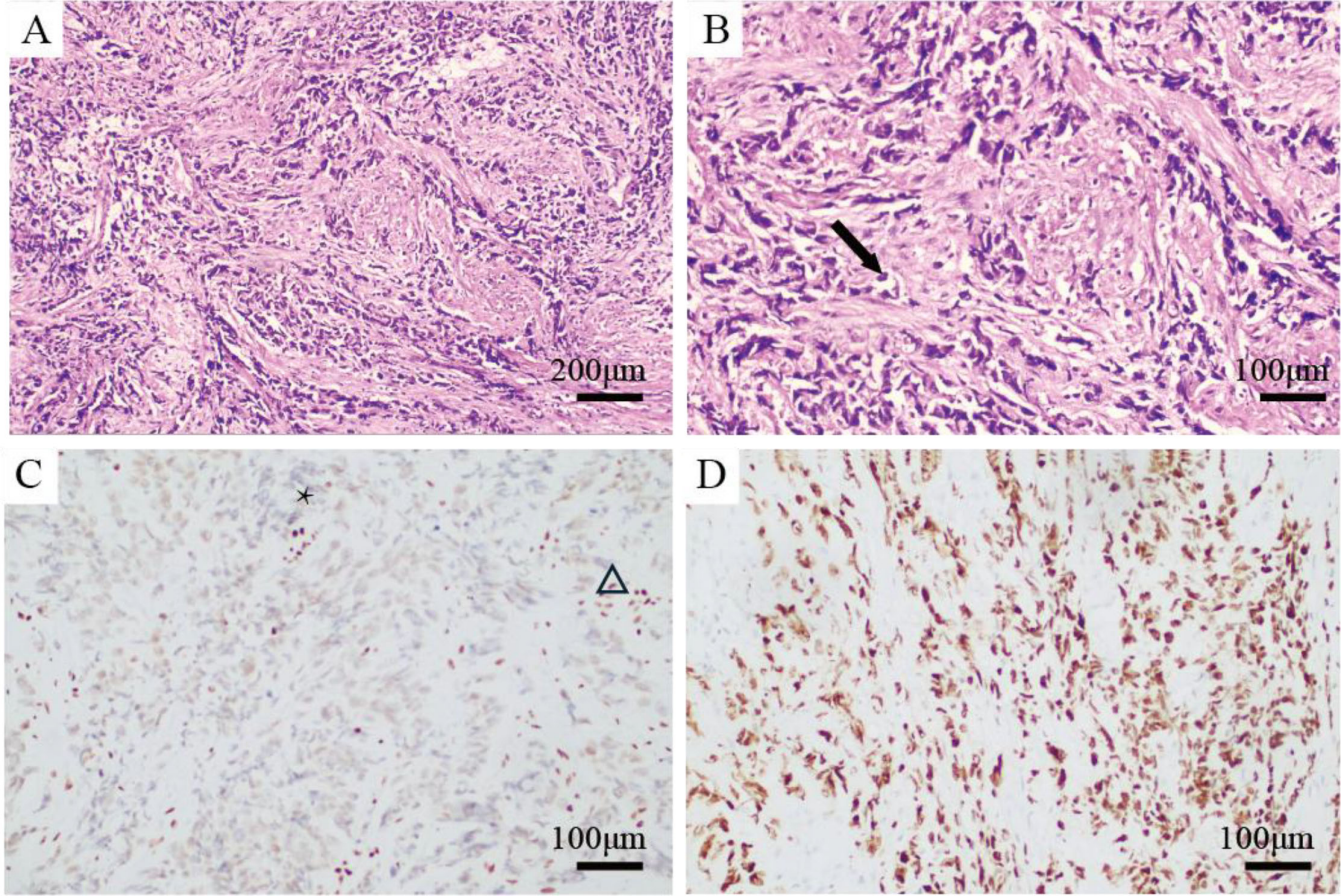
Hematoxylin-eosin (HE) and immunohistochemical staining of the patient. **(A)** H&E Staining (×100): The tumor shows diffuse growth and lacks glandular or squamous structures. **(B)** H&E Staining (×200): The tumor cells exhibit significant atypia, including pleomorphism and frequent mitotic figures (arrow). **(C)** SMARCA4/Brg1 (×200): Brg1 expression in tumor cell nuclei is completely absent (*), while control cells in the stroma, such as endothelial cells, show positive nuclear staining (△). **(D)** Ki-67 (×200): The proliferation index is approximately 70%.

**Figure 2:**
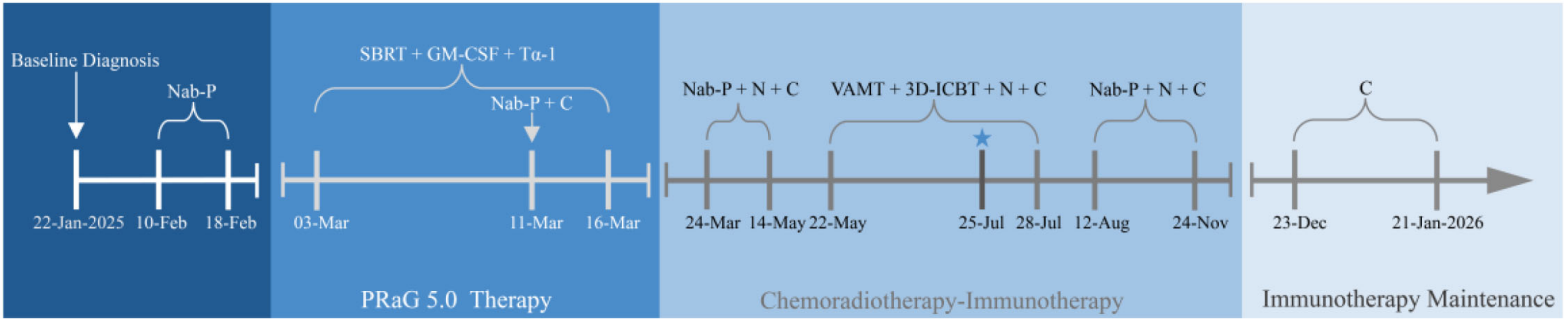
Treatment timeline. Nab-P, Nab-paclitaxel. N, Nedaplatin. C, Cadonilimab; Tα-1, Thymosin α1; SBRT, Stereotactic Body Radiotherapy; VMAT, Volumetric Modulated Arc Therapy; 3D-ICBT, Three-dimensional intracavitary brachytherapy; ★, Grade III neutropenia (lowest level 0.58 × 10^9^/L) occurred on July 25, 2025.

The patient, a 38-year-old female, was admitted in January 2025 with a history of irregular vaginal bleeding and lower abdominal pain lasting more than three months. On January 20, 2025, the enhanced CT scan of the chest and abdomen revealed a large cervical mass (9.8×9.8 cm) invading the rectum, bladder, small intestine, and right adnexa, along with multiple enlarged lymph nodes in the retroperitoneum, para-aortic region, bilateral iliac vessels, and left neck. On January 22, 2025, a biopsy of the cervical lesion showed CEA (–), CK (+ in a minority), P16 (+), P63 (–), Ki-67 (~70%), ER (–), PR (–), Vimentin (+ in partial cells), CK7 (–), CK20 (–), Pax8 (–), INI1 (+), and SMARCA4/Brg1 (–) (**Figure 1**). Due to limited tissue after these tests, PD-L1, MSI/MMR and tumor mutational burden status could not be assessed. Based on imaging and pathology findings, a diagnosis of SMARCA4-deficient undifferentiated cervical tumor, FIGO stage IVB, was established. The patient developed left hydronephrosis due to tumor invasion of the left ureter at its pelvic segment, leading to acute kidney injury (creatinine increased to 691.2μmol/L). Following emergency nephrostomy, the creatinine level decreased. Given the patient’s incomplete renal function recovery and poor physical condition (ECOG score of 2), nab-paclitaxel monotherapy (100mg) was administered on February 10th, 12th, and 18th, 2025, aiming for early tumor control. On February 20th, 2025, creatinine levels returned to normal, and the patient’s physical condition improved (ECOG score of 1). After a multidisciplinary team (MDT) discussion involving gynecologic oncology, radiation oncology, medical oncology, urology, pathology, and imaging (radiology) departments, and respecting the patient’s wishes, to rapidly and effectively control the large tumor, we employed the regimen of “PRaG 5.0 combined with sequential chemotherapy and chemoimmunotherapy” (**Figure 2**).

**Figure 3:**
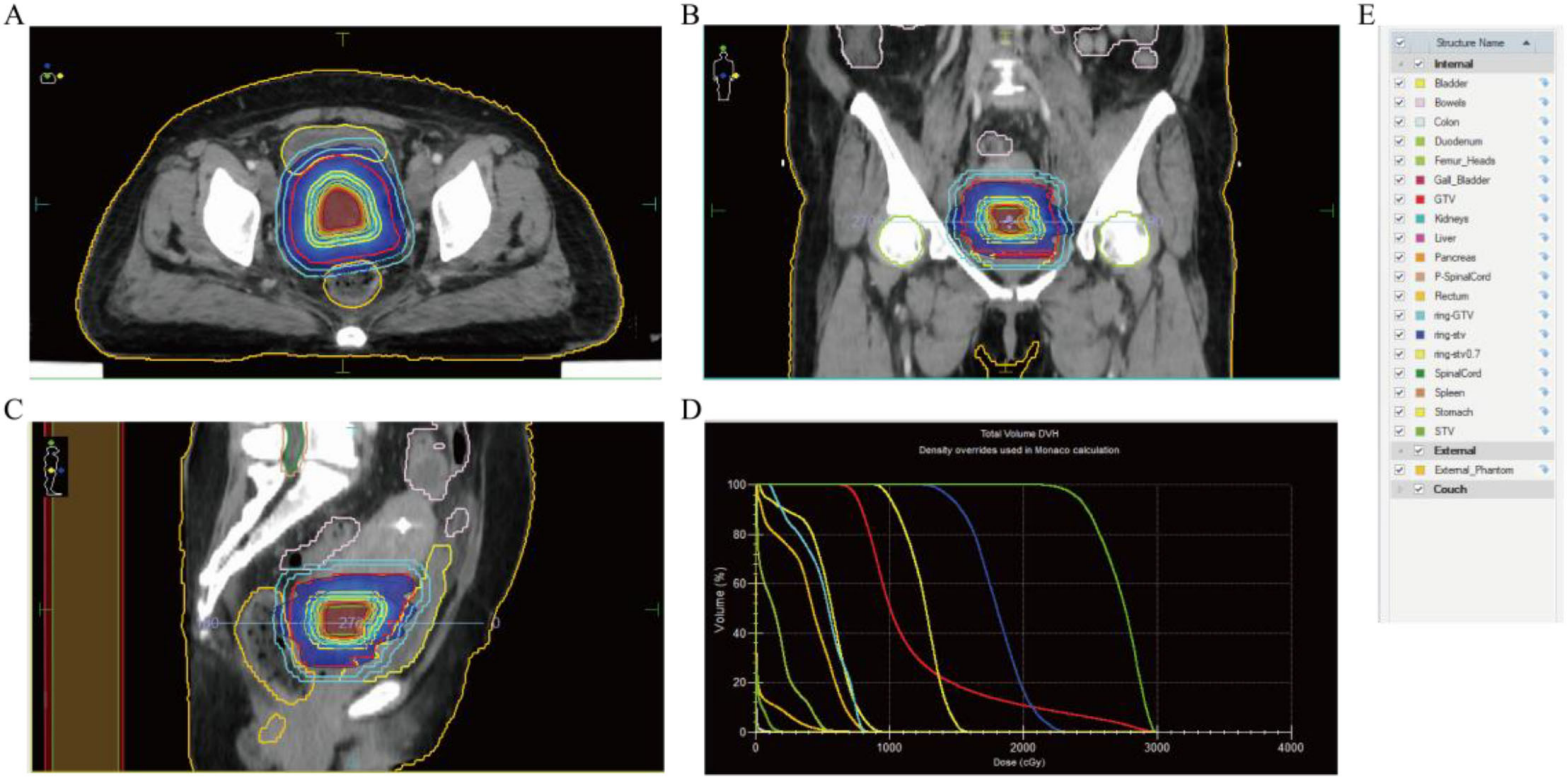
SBRT target volumes and dosimetry. GTV (Gross Target Volume) represents the overall anatomical range of the visible primary cervical tumor and metastatic sites on the CT images of this treatment plan, with a volume of 226.16 cm^3^. STV (Simultaneous Integrated Boost Target Volume): within the GTV, the core area most likely to be rich in active tumor cells determined by clinicians based on imaging characteristics, volume 13.16 cm^3^. **(A)** The radiotherapy target volume for the cervical lesion (axial view). **(B)** Radiotherapy target volume for the cervical metastases (coronal view). **(C)** Radiotherapy target volume for the cervical metastases (sagittal view). **(D)** Dose-volume histogram of radiotherapy plan. **(E)** List of organs at risk and target volume structures in the radiotherapy plan. Nevertheless, the bladder appeared flattened on the planning CT (3A) due to direct compression by the large cervical tumor, rather than inadequate filling. Bladder filling was monitored throughout radiotherapy using image guidance.

**Figure 4:**
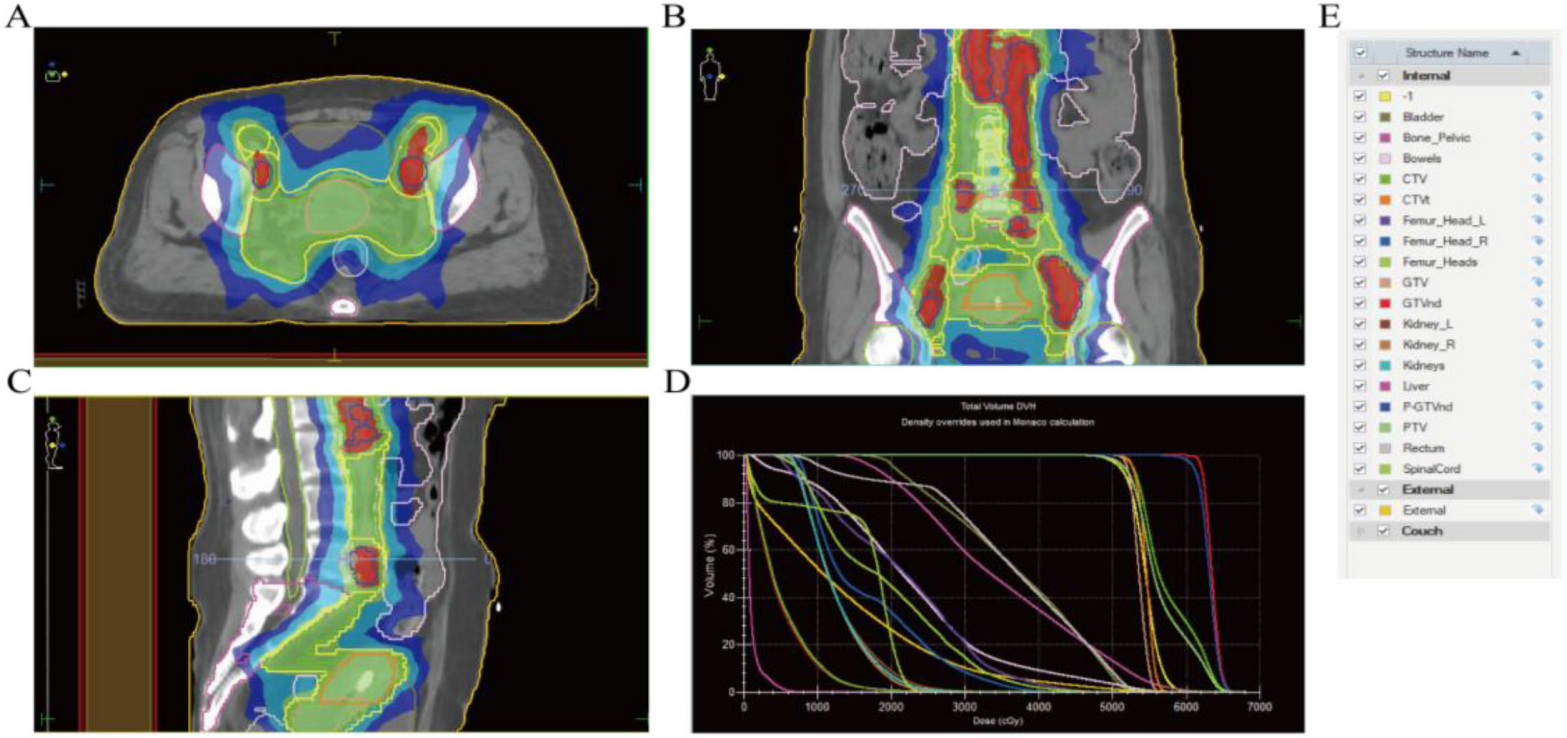
VMAT radiotherapy planning. **(A)** Radiotherapy target volumes for the primary cervical lesion and regional metastatic lymph nodes (axial view). **(B)** Target volumes for the primary cervical lesion and regional metastatic lymph nodes (coronal view). **(C)** Target volumes for the primary cervical lesion and regional metastatic lymph nodes (sagittal view). **(D)** Dose-volume histogram of the radiotherapy plan. **(E)** List of organs at risk and target volume structures in the radiotherapy plan. The superior border of the target volume was set at the upper margin of the L1 vertebral body to adequately cover the lymph nodes.

**Figure 5:**
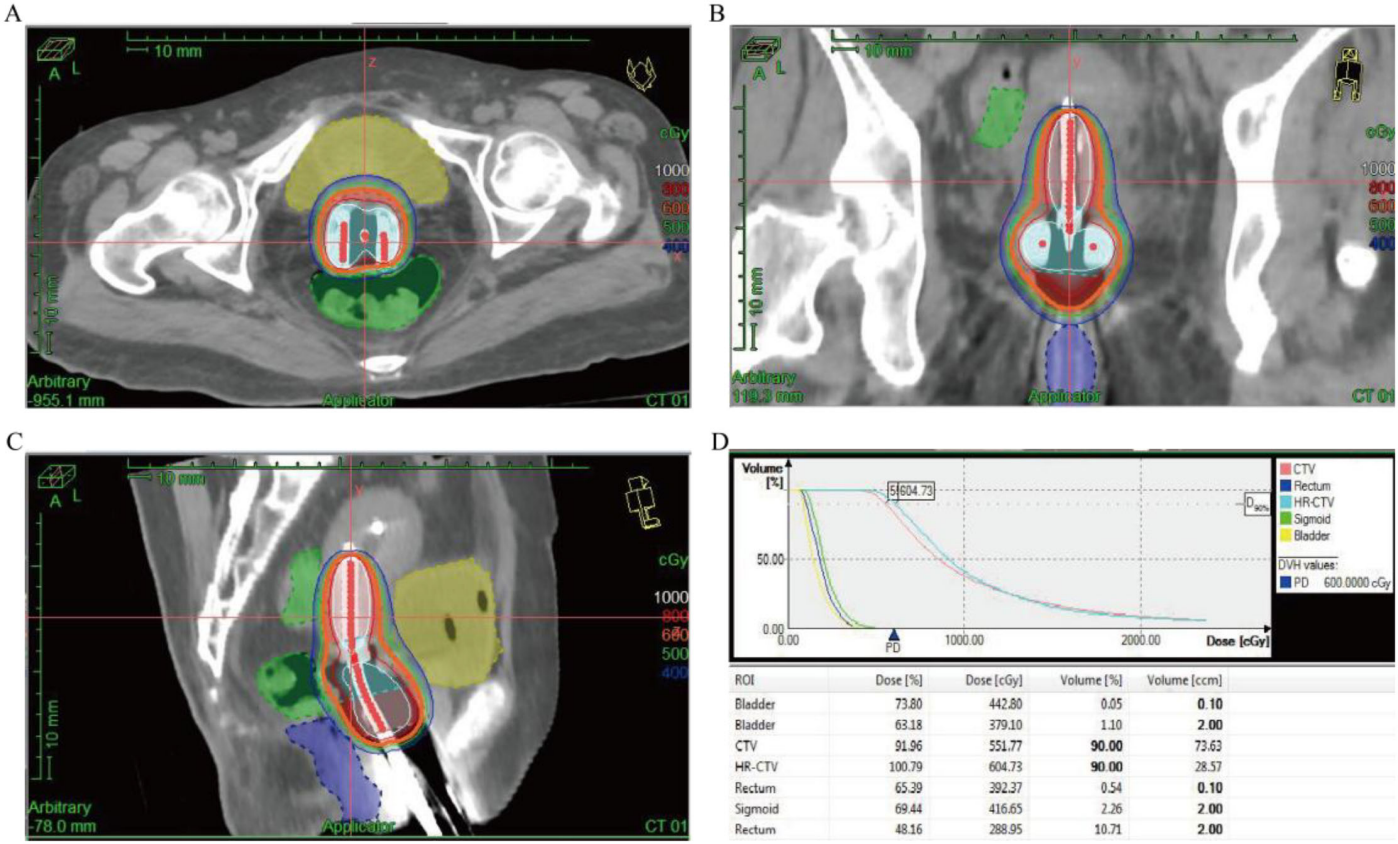
3D-ICBT for the cervical lesion. **(A)** Target volume for 3D-ICBT (axial view). **(B)** Dose distribution for 3D-ICBT (coronal view). **(C)** Dose distribution for 3D-ICBT (sagittal view). **(D)** Dose-volume histogram of 3D-ICBT plan.

**Figure 6:**
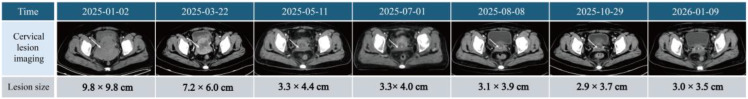
Serial contrast-enhanced abdominal CT images showing changes in the cervical lesion during treatment (arrows indicate the cervical lesion). In the CT images, the bladder, which varies in size depending on the filling status at the time of imaging, is located superior to the lesion. The rectum, which also varies in size due to distension from filling status, is located inferior to the lesion.

**Figure 7:**
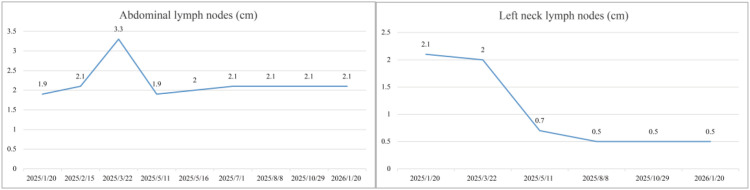
Time course of short-axis diameters of abdominal lymph nodes and left neck lymph nodes. Both showed progressive reduction during treatment.

**Figure 8:**
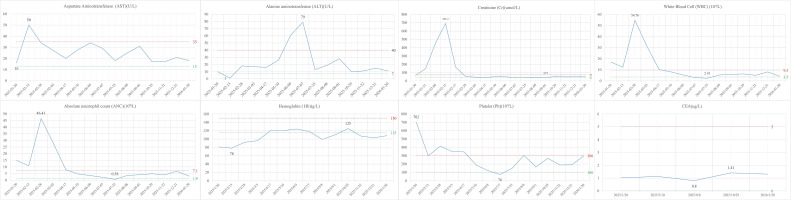
Changes in hematological indexes. The changes in the following hematological indexes during the treatment are shown: Aspartate Aminotransferase, Alanine aminotransferase, Creatinine, White blood cells, Absolute neutrophil count, Hemoglobin, Platelet, and CEA. Red lines represent the upper limits of normal and green lines represent the lower limits of normal, and the blue lines represent changes in patient indexes.

Specific treatment process: From March 3rd to March 5th, 2025, the cervical lesions underwent SBRT (24Gy/3Fx) (The patient followed a standardized bladder filling protocol (500–800 mL water, 1-hour holding) before CT simulation) (**Figure 3**), concurrently with GM-CSF (200μg/day × 7 days) and Thymosin α1, 1.6 mg per dose, administered three times a week. On March 11th, 2025, nab-paclitaxel 100 mg chemotherapy combined with cadonilimab 375 mg immunotherapy was administered. Two weeks later (March 22nd), the follow-up CT scan showed the tumor size to be 7.2×6.0 cm, and the efficacy evaluation indicated stable disease (SD). From March 2025 to May 2025, three cycles of nab-paclitaxel 400 mg + nedaplatin 100 mg + cadonilimab 375 mg were administered. On May 11th, a follow-up CT scan showed the tumor size to be 4.4×3.3 cm, and the efficacy evaluation showed PR. From May 22, 2025 to July 2, 2025, radical VMAT radiotherapy (P-GTVnd: 60.2 Gy/28 Fx, PTV: 50.4 Gy/28 Fx) was delivered to the primary lesion and the drainage area of metastatic lymph nodes (**Figure 4**). From June 25, 2025 to July 28, 2025, 3D-ICBT (CTV: 30 Gy/5 Fx) was performed (**Figure 5**). During the radiotherapy period, concurrent chemotherapy with nedaplatin 50 mg was administered on June 10, 2025, June 17, 2025, June 24, 2025, and July 1, 2025. Cadonilimab 250 mg immunotherapy was administered on June 10 and July 1, 2025. On August 8, 2025, a follow-up CT scan showed the cervical lesion measuring 3.9×3.1 cm, with an efficacy evaluation of PR. From August to December 2025, the patient received five cycles of nab-paclitaxel 400 mg, nedaplatin 100 mg, and cadonilimab 375 mg. On October 29, 2025, a follow-up abdominal CT scan showed a cervical lesion measuring 3.7×2.9 cm, with a PR response. From December 2025 to January 2026, the patient received 2 cycles of cadonilimab 375 mg for immunotherapy maintenance (**Figure 6**). Throughout the treatment period, serial imaging (contrast-enhanced CT for retroperitoneal/pelvic nodes, ultrasound for neck nodes) showed progressive reduction of all metastatic lymph nodes (**Figure 7**). Renal function normalized after nephrostomy and remained stable. Tumor biomarkers (CEA) were within normal limits, as were other hematological parameters (**Figure 8**). The only grade ≥3 adverse event was the grade III neutropenia (nadir 0.58×10^9^/L on July 25, 2025), which resolved with prophylactic granulocyte colony-stimulating factor (PEG-rhG-CSF) support. No other grade 3-4 toxicities occurred. To date, the patient’s PFS has exceeded 12 months.

## Discussion

SMARCA4, a tumor suppressor gene (6), encodes BRG1 protein, a core subunit of the SWI/SNF chromatin remodeling complex (7). SMARCA4-deficient undifferentiated carcinoma is frequently observed in young cancer patients (8); it is characterized by high invasiveness, early metastasis (9, 10), and primary chemotherapy resistance (11, 12). These features result in a very poor prognosis and a lack of standard treatment options (13–15). At initial diagnosis, the patient presented with a massive tumor and extensive lymph node metastasis, consistent with previously reported clinical features of high malignancy and high metastatic potential.

To rapidly alleviate patients’ symptoms, we implemented a treatment strategy of “PRaG5.0 combined with chemotherapy followed by chemoimmunotherapy”. However, due to acute kidney injury and active bleeding at initial presentation, a short bridging course of nab-paclitaxel monotherapy (three doses) was chosen for its safety, lack of nephrotoxicity, and bridging efficacy (16), allowing renal recovery before initiating the PRaG5.0−based regimen. The PRaG5.0 regimen, as a novel immunoradiotherapy combination, comprises core components: SBRT, immune checkpoint inhibitors (ICIs), GM-CSF, and thymosin α1 (5). The mechanism of action of this regimen includes the following specific steps: First, SBRT induces immunogenic cell death of tumor cells, thereby releasing tumor antigens (17, 18). Second, GM-CSF promotes the proliferation of dendritic cells (DCs), enhancing their ability to uptake, process, and present tumor antigens (19, 20). It should be noted that GM-CSF has a dual role in the tumor microenvironment and may also induce immunosuppressive myeloid cells under certain conditions (21). To mitigate this risk, the PRaG5.0 protocol employs a short, pulsed course of GM-CSF (7 days) immediately after SBRT and combines it with thymosin α1, a classic immunomodulator, promotes the proliferation and maturation of precursor T cells, reshaping the tumor microenvironment (22, 23), to actively counteract potential immunosuppression (24). Finally, cadonilimab, a PD-1/CTLA-4 bispecific antibody, alleviates T cell suppression, thereby enabling activated effector T cells to recognize and eliminate tumor cells more effectively.

The effectiveness of the PRaG regimen has been confirmed by multiple clinical studies. A phase II study showed that the median overall survival of the PRaG regimen in refractory solid tumors was 10.5 months, and some patients resistant to ICIs achieving complete remission (25). A retrospective study of MSS/pMMR metastatic colorectal cancer showed that median progression-free survival in the PRaG group was 4.5 months, which was significantly better than the PD-1 inhibitor combined with chemotherapy group (3.1 months) or the combined TKI group (3.4 months, P<0.05) (26). Furthermore, the PRaG regimen has shown significant efficacy in the second-line treatment of tumors such as gastric cancer (27), pancreatic cancer (28), and triple-negative breast cancer (29). In the present case, the PRaG5.0 regimen was adopted, with short-course SBRT as the core radiotherapy component. The favorable response to SBRT may be partially attributed to the inherent radiosensitivity of poorly differentiated tumors: high proliferative activity (Ki-67 ~70%) enriches G2/M cells, enhancing low-dose hyper-radiosensitivity (30), although hypoxic microenvironments are common, radiotherapy induces reoxygenation partly via acute inflammation (31). Additionally, the loss of SMARCA4/BRG1 impairs DNA damage repair, thereby further enhancing sensitivity to ionizing radiation (32). Yokoe et al. (33) reported a case of SMARCA4-deficient cervical cancer that remained recurrence-free for more than one year after R0 resection followed by adjuvant radiotherapy. Liang et al. (34) described a case of thoracic SMARCA4-deficient undifferentiated tumor in which radiotherapy combined with chemoimmunotherapy achieved an overall survival exceeding 33 months. Therefore, We administered SBRT on the large cervical lesion (total volume 226.16 cm³) in 3 fractions. The core area within the lesion (13.16 cm³) received a focused high dose of 24.3 Gy (peak 29.89 Gy), while the remaining part of the lesion received 7.5 Gy. The mean doses to the bladder and rectum were controlled at 5.40 Gy and 4.02 Gy, respectively. This approach ensured that the surrounding normal tissues were protected to the maximum extent while effectively killing the tumor. Due to the patient’s renal insufficiency, the initial PRaG5.0 regimen was combined with nab-paclitaxel chemotherapy. After the patient’s renal function improved, sequential cadonilimab immunotherapy was administered in combination with nedaplatin and albumin-bound paclitaxel chemotherapy. This regimen resulted in significant relief of the patient’s vaginal bleeding symptoms within two weeks following the initial PRaG5.0 combined chemotherapy. The patient’s PFS exceeded 12 months, a duration far surpassing the previously reported median overall survival (mOS) of less than 6-7 months for this tumor type (2, 35, 36).

Currently, there is a lack of standard treatment options for SMARCA4-deficient tumors, although recent studies indicate that immunotherapy combined with chemotherapy may have advantages in this type of tumor (37, 38). Shi et al. reported that the PFS of patients with advanced SMARCA4-deficient thoracic tumors who received chemotherapy combined with immunotherapy was 6.90 months, which was better than the 5.10 months of chemotherapy combined with bevacizumab (p<0.05) (39); Liu et al. further confirmed that in thoracic SMARCA4-deficient tumors, the mOS of chemotherapy combined with immunotherapy significantly exceeded that of chemotherapy combined with anti-angiogenic therapy (21.67 vs. 8.80 months, P = 0.02) (40). These studies show that in SMARCA4-deficient tumors, the efficacy of “chemotherapy + immunotherapy” may appear superior to that of “chemotherapy + anti-angiogenic therapy”. Regarding the selection of immune checkpoint inhibitors, Xu et al. reported a case of a patient with SMARCA4-deficient cervical cancer who received cadonilimab combined with paclitaxel and cisplatin, with PFS exceeding 7 months (41). Another case of a patient with lung adenocarcinoma and pancreatic metastases who received cadonilimab combined with local radiotherapy achieved a PFS of 11 months (42). Furthermore, the COMPASSION-16 phase III study confirmed that cadonilimab combined with chemotherapy can significantly prolong PFS and OS in cervical cancer patients (43). At the initial diagnosis of this patient, active bleeding was a contraindication for bevacizumab (44), and PD−L1, microsatellite instability (MSI) and mismatch repair (MMR) protein expression were not tested due to insufficient tumor specimens. Nevertheless, relevant studies suggest that SMARCA4/BRG1 deficiency leads to genomic instability by impairing DNA double−strand break repair, which may enhance tumor immunogenicity and sensitivity to immune checkpoint inhibitors regardless of PD−L1 expression level (45, 46). Longo et al. stated that the efficacy of immunotherapy in SMARCA4−deficient undifferentiated tumors (SMARCA4−UT) appears to be independent of PD−L1 expression (47). Liu et al. reported a patient with PD−L1−low (TPS < 1%) thoracic SMARCA4−UT who achieved a partial response with first−line camrelizumab−containing immunotherapy plus chemotherapy (48). Similarly, in the case reported by Xu et al., the PD-L1 status was not tested (unknown) (41). Regarding MMR status in SMARCA4-deficient tumors, most reported cases are pMMR/MSS (35, 36, 49), However, a minority of cases may exhibit MMR abnormalities. Elzamly et al. (50) analyzed MMR status in 15 out of 22 cases of esophageal SMARCA4-deficient undifferentiated carcinoma and clearly identified one case (7%) with dMMR. Therefore, determining MMR status remains important, even in this rare entity. The inability to perform this test due to limited tissue is a limitation of this study. Therefore, cadonilimab was chosen as the immunotherapy drug instead of bevacizumab. The standard cadonilimab dose is 10 mg/kg every 3 weeks (43), but due to the patient’s poor initial condition, we reduced the dose to 6.7 mg/kg (375 mg), referencing the dose-toxicity correlation from COMPASSION-01 (6 mg/kg Q2W: 6.7% grade ≥3 irAEs) (51) to COMPASSION-16 (10 mg/kg Q3W: 82% grade ≥3 TRAEs). Later, during the VAMT + 3D-ICBT phase, we further reduced the dose to 250 mg as a prophylactic measure, based on evidence that dual PD-1/CTLA-4 blockade raises pneumonitis (10% vs. 3%) (52), and renal adverse events (53), and that cadonilimab itself has significant hematologic toxicity, which may be further aggravated by large-field pelvic radiotherapy (54), we prophylactically reduced the dose to 250 mg (≈4.5 mg/kg). Notably, even with this dose reduction, the patient developed grade III neutropenia (nadir 0.58×10^9^/L), underscoring the necessity of dose attenuation. After radiotherapy, the dose was restored to 375 mg. Regarding the chemotherapy drug, nedaplatin was chosen instead of cisplatin due to the patient’s renal insufficiency. Previous studies have shown that nedaplatin is non-inferior to cisplatin in terms of in cervical cancer, with lower nephrotoxicity and gastrointestinal reactions (55). As a low-nephrotoxic cisplatin derivative (56), it is more suitable for this patient with impaired renal function.

The main adverse reaction in this patient was a hematologic toxicity. Grade III neutropenia occurred concurrently with VMAT radiotherapy. Although the initial SBRT delivered a high single dose, the target area was highly concentrated in the tumor core, with limited impact on the surrounding hematopoietic bone marrow; while the subsequent VMAT radiotherapy covered the primary lesion and regional lymph node drainage areas, inevitably irradiating large active hematopoietic areas such as the pelvis (57). However, after standard PEG-rhG-CSF support, the white blood count recovered rapidly, which did not affect subsequent treatment. The patient’s creatinine level remained normal after nephrostomy and did not increase even with subsequent nedaplatin combined with chemotherapy. Regarding the risk of fistula, literature reports an incidence of 0−13% following cervical SBRT (58), and the addition of bevacizumab significantly increases this risk (3−year fistula rate: 27.0% in the bevacizumab group vs. 3.0% in the radiotherapy−alone group; HR 4.76) (59). The patient did not receive bevacizumab, which further reduced the risk. No fistula or other radiation−related complications occurred in this patient.

Due to limited tissue, PD−L1, MSI/MMR, and TMB status could not be assessed. As a single case report, these findings require validation in larger studies. Nevertheless, this report demonstrates the potential of the PRaG5.0−based strategy for massive SMARCA4−deficient cervical tumors. Future prospective studies with comprehensive biomarker analysis—including PD−L1, MSI/MMR, and TMB—are warranted to further confirm its efficacy and safety. Moreover, as a single case report without a comparator, the specific contribution of the PRaG5.0-based regimen cannot be isolated from the multimodal treatment. Given the lack of established standard therapy for this rare entity, our findings are hypothesis-generating and require validation in larger, controlled cohorts.

## Conclusion

This case report presents the innovative use of a strategy of PRaG5.0 combined with chemotherapy followed by sequential immunochemotherapy to treat a stage IVB massive SMARCA4-deficient cervical tumor, achieving sustained tumor regression with good tolerability. Notably, the patient’s PFS exceeded 12 months. These results demonstrate that the PraG5.0 combined with chemotherapy followed by a sequential immunochemotherapy is a novel treatment option that merits further investigation for massive SMARCA4-deficient cervical tumors.

## Data Availability

The original contributions presented in the study are included in the article/supplementary material. Further inquiries can be directed to the corresponding authors.
